# Characterization of sub-nuclear changes in *Caenorhabditis elegans *embryos exposed to brief, intermediate and long-term anoxia to analyze anoxia-induced cell cycle arrest

**DOI:** 10.1186/1471-2121-6-47

**Published:** 2005-12-20

**Authors:** Vinita A Hajeri, Jesus Trejo, Pamela A Padilla

**Affiliations:** 1 Department of Biological Sciences, University of North Texas, Denton, TX, 76203, USA

## Abstract

**Background:**

The soil nematode *C. elegans *survives oxygen-deprived conditions (anoxia; <.001 kPa O_2_) by entering into a state of suspended animation in which cell cycle progression reversibly arrests. The majority of blastomeres of embryos exposed to anoxia arrest at interphase, prophase and metaphase. The spindle checkpoint proteins SAN-1 and MDF-2 are required for embryos to survive 24 hours of anoxia. To further investigate the mechanism of cell-cycle arrest we examined and compared sub-nuclear changes such as chromatin localization pattern, post-translational modification of histone H3, spindle microtubules, and localization of the spindle checkpoint protein SAN-1 with respect to various anoxia exposure time points. To ensure analysis of embryos exposed to anoxia and not post-anoxic recovery we fixed all embryos in an anoxia glove box chamber.

**Results:**

Embryos exposed to brief periods to anoxia (30 minutes) contain prophase blastomeres with chromosomes in close proximity to the nuclear membrane, condensation of interphase chromatin and metaphase blastomeres with reduced spindle microtubules density. Embryos exposed to longer periods of anoxia (1–3 days) display several characteristics including interphase chromatin that is further condensed and in close proximity to the nuclear membrane, reduction in spindle structure perimeter and reduced localization of SAN-1 at the kinetochore. Additionally, we show that the spindle checkpoint protein SAN-1 is required for brief periods of anoxia-induced cell cycle arrest, thus demonstrating that this gene product is vital for early anoxia responses. In this report we suggest that the events that occur as an immediate response to brief periods of anoxia directs cell cycle arrest.

**Conclusion:**

From our results we conclude that the sub-nuclear characteristics of embryos exposed to anoxia depends upon exposure time as assayed using brief (30 minutes), intermediate (6 or 12 hours) or long-term (24 or 72 hours) exposures. Analyzing these changes will lead to an understanding of the mechanisms required for initiation and maintenance of cell cycle arrest in respect to anoxia exposure time as well as order the events that occur to bring about anoxia-induced cell cycle arrest.

## Background

Oxygen deprivation is an environmental condition organisms may encounter in their natural habitat, thus mechanisms evolved to respond to and survive oxygen deprivation. Hypoxia and anoxia are both terms used to describe oxygen deprivation. Hypoxia has been defined in several ways including: 1. When O_2 _deprivation limits electron transport, 2. A state of reduced O_2 _availability or decreased oxygen partial pressures (pO_2_), 3. When a decrease in O_2 _results in an abolishment or reduction of functions in organs, tissues or cells. Anoxia is sometimes referred to as a state of "severe hypoxia" yet the term anoxia typically describes the absence of detectable O_2 _in either the tissue or the environment that an organism is exposed to [1-3].

In regards to human health, oxygen deprivation is central to the pathology of several diseases including myocardial infarction, pulmonary disease, and solid tumor progression. Oxygen deprivation can also cause severe cellular damage as a result of trauma due to blood loss, suffocation or drowning. Thus, it is of interest to identify the molecular responses to oxygen deprivation. Several model systems are used to understand the physiological response organisms have to oxygen deprivation [4,5]. For example, anoxia tolerant organisms are capable of decreasing energy usage by stopping non-essential cellular functions, maintain stable and low permeability of membranes, and produce ATP by glycolysis [6]. However, the sub-cellular response to oxygen deprivation, in developing embryos, is less understood.

Oxygen deprivation influences the growth, development, and behavior of the soil nematode *Caenorhabditis elegans*. For example, *C. elegans *exposed to anoxia (<.001 kPa O_2_) in laboratory culture conditions displays the remarkable characteristic of suspended animation in which embryonic development and cell cycle progression arrests and post-embryonic nematodes arrest development, feeding, movement, and in the case of adults, do not lay eggs [7,8]. These arrested biological processes in the nematode resume upon re-exposure to normoxia. Several organisms are capable of arresting embryonic development and cell cycle progression in response to oxygen deprivation [9-11]. Blastomeres of *C. elegans *and *D. melanogaster *embryos exposed to anoxia arrest during interphase, some stages of mitosis, predominately prophase and metaphase, but not anaphase [7,10]. *D. melanogaster *embryos exposed to hypoxia arrest in interphase and the metaphase stage of mitosis [12-14]. In comparison, blastomeres of zebrafish embryos exposed to anoxia arrest during interphase [11]. Analysis of interphase blastomeres of *C. elegans*, zebrafish and *Drosophila *embryos exposed to anoxia indicates that the chromatin appears condensed and is not uniformly distributed throughout the nucleus [7,10,11]. Thus, not only is the phenomena of anoxia-induced suspended animation conserved but some of the cellular responses and mechanisms involved with suspended animation are evolutionarily conserved.

The use of genetic model systems has increased our understanding of the mechanisms regulating oxygen deprivation sensing and survival [15-20]. For example, in *C. elegans*, an RNA interference (RNAi) genomic screen provided evidence that the spindle checkpoint proteins, SAN-1 and MDF-2, homologous to the yeast MAD3 and MAD2 spindle checkpoint proteins respectively, are active in the early embryo and are required for anoxia-induced suspended animation [21-23]. Additionally, the *Drosophila *spindle checkpoint protein Mps1, is required for hypoxia-induced arrest [24]. Thus, the oxygen-deprivation induced cell cycle arrest by the spindle checkpoint proteins is likely a conserved process. The spindle checkpoint components monitor kinetochore-microtubule attachment and tension and delay the metaphase to anaphase transition until chromatids are adequately attached to microtubules [25-27]. The requirement of spindle checkpoint proteins for oxygen deprivation arrest supports the idea that an oxygen-sensing pathway regulates the spindle checkpoint components, however the mechanism for such a pathway is not understood.

Analysis of the cellular structures in blastomeres of embryos deprived of oxygen will provide insight into how oxygen-deprivation tolerant organisms, such as *C. elegans*, respond to and survive oxygen deprivation. The *C. elegans *embryo exposed to 24-hours of anoxia displays specific distinguishing features such as dephosphorylation of cell cycle regulated proteins including histone H3 and proteins recognized by the MPM-2 antibody, kinetochore rearrangements, and astral microtubule depolymerization [7,28]. In this report, we expand the analysis of the sub-cellular changes in *C. elegans *embryos exposed to anoxia for various periods of time. The cellular structures examined include: nuclear localization of chromatin, phosphorylation of histone H3, spindle microtubule structure, and SAN-1 localization pattern. Also, we demonstrate that the cellular-structures differ depending upon anoxia exposure time. We suggest that microtubule and SAN-1 alterations are involved with the arrest of metaphase blastomeres, because we find that in embryos exposed to brief periods of anoxia the spindle microtubules depolymerize, SAN-1 localization to the kinetochore is altered and SAN-1 is required for brief periods of anoxia. The work presented here raises the idea that analysis of cellular structures can be used as hallmarks of oxygen deprivation exposure time. Moreover, identifying the temporal order of cellular changes in response to anoxia yields a framework to differentiate the specific mechanisms required for the establishment or maintenance of anoxia-induced suspended animation.

## Results

*C. elegans *embryos arrest development and cell cycle progression at the time the environment turns anoxic, indicating that one of the first responses to anoxia is developmental and cell cycle arrest. Embryos remain viable in this arrested state for at least three days. Upon re-exposure to normoxic conditions, developmental and cell cycle progression resumes and the embryo develops into a fertile adult nematode [7]. We used cell biological techniques such as time-lapse microscopy and indirect immunofluorescence to determine if anoxia exposure time affects recovery and sub-nuclear structures in the embryo.

### Recovery time from anoxia is dependent upon anoxia exposure time

**Figure 1:**
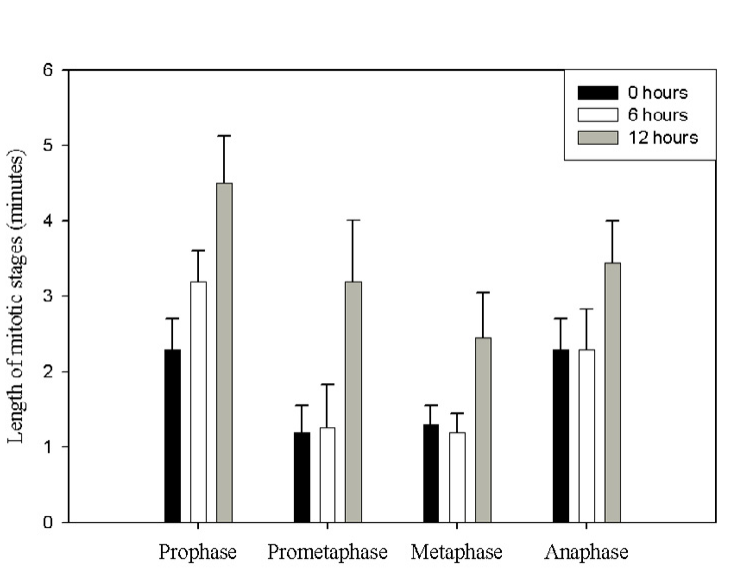
**Wild-type embryos removed from 12 hours of anoxia have a reduced rate of mitotic progression in comparison to wild-type embryos exposed to normoxic environments. **Mitotic progression in blastomeres of wild-type embryos exposed to normoxia (black bar), 6 hours of anoxia (white bar) and 12 hours of anoxia (grey bar) was assayed using a histone2B::GFP strain and DIC microscopy. The embryos exposed to anoxia were allowed to recover in air for 15 minutes before analysis. The average time to condense chromosomes (prophase), move condensed chromosomes to the metaphase plate (prometaphase), align chromosomes along the metaphase plate (metaphase), and segregate chromosomes (anaphase) was determined for 10 embryos for each condition. Error bar equals standard deviation. For the 12 hour anoxia exposure data p < .001 when compared to normoxic control. For the 6 hour anoxia exposure data p < .002 for prophase only and data was not significantly different in comparison to normoxia for the other mitotic stages. Error bars represent standard deviation.

To investigate the recovery of *C. elegans *blastomeres from cell cycle arrest, embryos were exposed to 6 or 12 hours of anoxia, removed from anoxia, allowed to recover in air for 15 minutes and then visualized using time-lapse microscopy. Briefly, the strain AZ212 (*pie-1*::Histone H2B::GFP) [29] was used to visualize mitotic progression by analyzing chromosome condensation and segregation. Mitotic progression was monitored using differential interference contrast (DIC) and fluorescent microscopy. Images were collected and analyzed to determine the time blastomeres were at specific stages of mitosis. Figure 1 shows that young embryos exposed to 12 hours of anoxia and allowed to recover in air for 15 minutes take longer to progress through stages of mitosis in comparison to embryos exposed to either a normoxic environment or 6 hours of anoxia. Embryos exposed to normoxia progress through mitosis on average 7.1 ± 1.4 minutes (N = 10 blastomeres), which is similar to data reported by others [22,30]. Embryos exposed to 6 hours of anoxia and allowed to recover in normoxia for 15 minutes progress through mitosis on average 8.0 ± 1.8 minutes (N = 10 blastomeres). However, blastomeres, of embryos exposed to 12 hours of anoxia and allowed to recover in normoxia for 15 minutes progress through mitosis on average 13.6 ± 2.6 minutes (N = 10 blastomeres; p < .001). The progression through each stage of mitosis was significantly different for blastomeres of embryos exposed to 12 hours of anoxia in comparison to those exposed to normoxia (Figure 1). Embryos exposed to 24 hours of anoxia required at least 30 minutes of normoxia recovery before mitotic progression was detected (data not shown). Taken together, these data demonstrate that anoxia exposure time can affect recovery from anoxia.

### Embryos exposed to anoxia contain blastomeres with chromosome alterations including condensation and chromosome alignment with the nuclear membrane

The observation that embryos recovering from 12 hours of anoxia initially progressed through mitosis at a slower rate in comparison to the embryos exposed to 6 hours of anoxia led to the hypothesis that the sub-cellular characteristics of embryos exposed to anoxia are a function of anoxia exposure time. To test this hypothesis embryos were exposed to anoxia for various time periods and indirect immunofluorescent assays were used to analyze sub-cellular nuclear structures. Specifically, embryos were exposed to 30 minutes, 6, 12, 24, or 72 hours of anoxia, fixed and stained with the DNA binding dye DAPI and antibodies to distinguish stages of mitosis and visualize sub-cellular nuclear structures. The distinction of cell cycle stages of blastomeres can be determined by using an antibody (mAb414) that recognizes the nuclear pore complex and the DNA binding dye DAPI [7,31]. Specifically, in *C. elegans *the mAb414 antibody is associated with the nuclear membrane during interphase, prophase and prometaphase and breaks down in metaphase and reforms with the nuclear membrane in telophase [7,31]. We used mAb414 and DAPI to analyze chromatin morphology and nuclear location in blastomeres of embryos exposed to anoxia.

**Figure 2:**
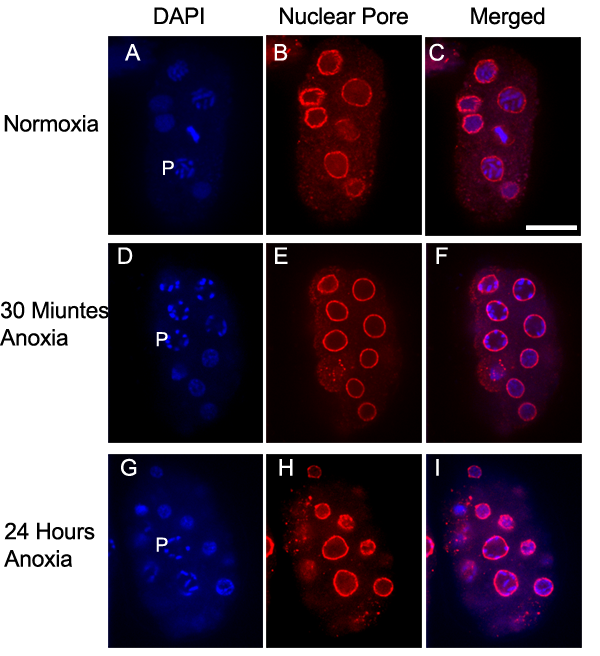
**Chromosomes of prophase blastomeres exposed to brief periods of anoxia align near the nuclear membrane. **Embryos were exposed to either normoxia (A-C) or 30 minutes of anoxia (D-F), or 24 hours of anoxia (G-I) collected, and stained with DAPI (A, D, G) and the mAb414 to recognize nuclear pore complex (B, E, H). Images were merged (C, F, I) to evaluate prophase chromosome location relative to the nuclear membrane. The letter P is to the left of prophase blastomeres. Scale bar is 10 μM.

Blastomeres containing condensed chromosomes that could be individually distinguished but were not located near or at the equatorial plate were classified as prophase blastomeres. Figure 2 suggests that the prophase blastomeres of embryos exposed to 30 minutes or 24 hours of anoxia contain chromosomes localized near the nuclear membrane whereas the normoxic control embryos contain prophase blastomeres with chromosomes throughout the nucleus. To further investigate the possibility that anoxia induces the prophase chromosomes to localize near the nuclear membrane we used spinning disk confocal microscopy to produce movies of the Z-stacked cross section images obtained from prophase nuclei of embryos exposed to either normoxia or anoxia. Prophase chromosomes localized throughout the nucleus of an embryo exposed to normoxia [see Additional file 1]. Whereas, prophase chromosomes localized near the nuclear membrane of embryos exposed to 30 minutes or 24 hours of anoxia [see Additional files 2 and 3]. We verified our method of identifying prophase blastomeres by using the anti-gamma tubulin antibody to detect centrosome position. Briefly, prophase blastomeres will contain duplicated centrosomes across from one another relative to the nucleus [32]. A representative prophase nucleus from embryos exposed to either normoxia or 30 minutes of anoxia are shown [see Additional files 4 and 5]. We saw a correlation of chromosomes that were condensed and individually distinct with centrosomes that were duplicated and across from one another, indicating that these blastomeres were indeed prophase blastomeres. The characteristic of chromosomes located near the nuclear membrane is a hallmark of arrested prophase blastomeres in that it is observed in all embryos exposed to anoxia for either brief periods of anoxia (30 minutes) or longer periods of anoxia (72 hours, data not shown).

**Figure 3:**
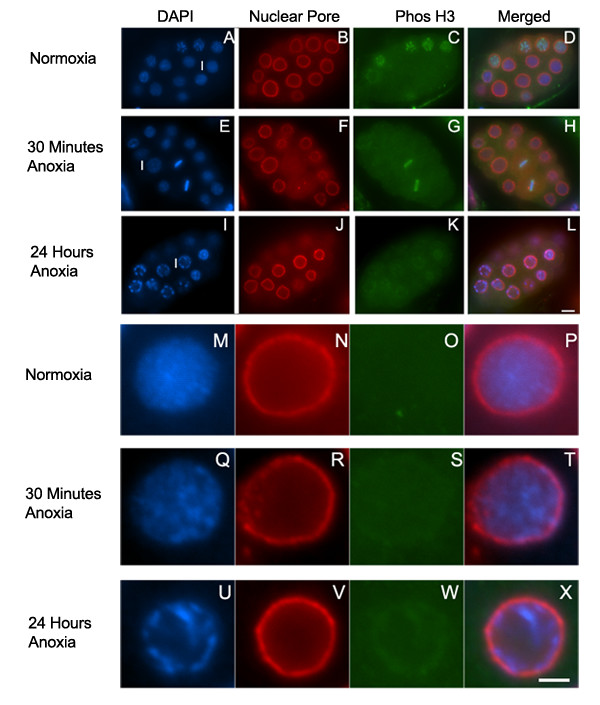
**Chromosomes of interphase blastomeres exposed to anoxia condense and align near the nuclear membrane. **Embryos were exposed to either normoxia (A-D, M-P), 30 minutes of anoxia (E-H, Q-T), or 24 hours of anoxia (I-L, U-X), collected, and stained with DAPI (A, E, I, M, Q, U), the mAb414 to recognize nuclear pore complex (B, F, J, N, R, V), and PhosH3, which recognizes phosphorylated Histone H3 (C, G, K, O, S, W). Images were merged (D, H, L, P, T, X) to analyze chromosome location relative to the nuclear membrane. In figures A, E, F, letter I is to the left of the interphase blastomere enlarged and shown in figures M-X respectively. Scale bar equals 5 μM for images A-L and 2 μm for images M-X.

It was previously determined that *C. elegans*, *Drosophila*, and zebrafish embryos exposed to 24 hours of anoxia contain interphase blastomeres with chromosomal DNA that is not uniformly distributed throughout the nucleus and instead appears condensed [7,10,11]. We were interested in determining if interphase blastomeres exposed to brief periods of anoxia showed chromatin condensation. Figure 3 shows that embryos exposed to 30 minutes of anoxia do contain blastomeres with condensed chromatin. The blastomeres containing chromosomes that were condensed but were not individually distinguished were scored as interphase blastomeres. We verified this method of identifying interphase blastomeres by staining with anti-gamma tubulin. We saw a correlation of chromatin that was condensed yet displayed no distinct individual chromosomes with centrosomes that were unduplicated or duplicated yet in close proximity to one another, indicating that these blastomeres were indeed interphase blastomeres (data not shown). We examined embryos exposed to 6, 12, 24 or 72 hours of anoxia and determined that in interphase blastomeres, increased chromatin condensation correlated with longer anoxia exposure times. Additionally, the condensed chromatin is localized near the nuclear membrane region rather than throughout the nucleus. Figure 3U shows a representative interphase blastomere of an embryo exposed to 24 hours of anoxia. Thus, chromatin condensation in interphase blastomeres is a characteristic of embryos exposed to anoxia, but the nuclear location of the chromatin is dependent upon anoxia exposure time.

To analyze the metaphase plate in embryos exposed to anoxia, embryos were subjected to various anoxia exposure times and stained with DAPI and DM1A, an antibody that recognizes microtubules. To determine if embryos exposed to anoxia contain metaphase blastomeres in which the metaphase plate size is altered, we measured the length (diameter of the metaphase plate) and the width of the metaphase plate in blastomeres of embryos, at similar developmental stages, exposed to either normoxic or anoxic conditions. The metaphase plate length and width was determined using the imaging software program Openlab 3.1.7. Table 1 shows that in comparison to normoxic control embryos, embryos exposed to 6 hours or more of anoxia had a significantly reduced metaphase plate length and embryos exposed to 12 hours of anoxia had a significantly increased metaphase plate width. This data supports the idea that the metaphase plate is altered in blastomeres of embryos exposed to anoxia. Taken together, these data demonstrate that anoxia affects chromosomes and these chromosome alterations are either detected after brief periods of anoxia, as is the case with prophase and interphase chromosome alterations, or after several hours of anoxia, as is the case with metaphase chromosomes.

**Table 1:** Metaphase plate and spindle structure in anoxic embryos

	Metaphase Plate		Metaphase
	Length ± SD (μm)	Width ± SD (μm)	Spindle Perimeter ± SD (μm)
Normoxia	4.33 ± .48	.96 ± .13	13.83 ± .85
Anoxia:			
30 minutes	3.92 ± .45	.87 ± .10	13.73 ± .87
6 hours	3.51 ± .19	1.12 ± .19	9.20 ± .74
12 hours	3.23 ± .10	1.24 ± .13	8.41 ± .25
24 hours	2.51 ± .30	1.26 ± .07	6.77 ± .63
72 hours	2.71 ± .18	1.25 ± .04	6.82 ± .37

For each assay at least 12 embryos were assayed.

SD is Standard Deviation

The p value was < .001 for metaphase plate length and metaphase spindle perimeter measurements for embryos exposed to 6 or more hours of anoxia and for metaphase plate width measurements for embryos exposed to 12 hours of anoxia.

### Histone H3 dephosphorylation is not an initial response to anoxia exposure

**Figure 4:**
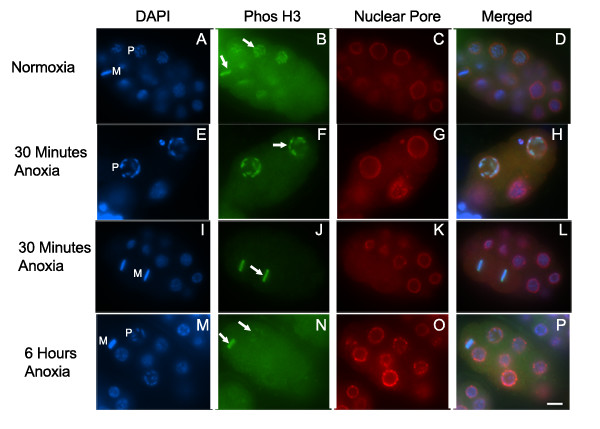
**Phosphorylated Histone H3 is phosphorylated in embryos exposed to brief periods of anoxia. **Embryos were exposed to either normoxia (A-D), 30 minutes of anoxia (E-L), or 6 hours of anoxia (M-P), collected, and stained with DAPI (A, E, I, M), PhosH3 recognizes phosphorylated Histone H3 (B, F, J, N), and the mAb414 to recognize nuclear pore complex (C, G, K, O). Merged image for each set is shown (D, H, L, P). PhosH3 is detected in prophase and metaphase blastomeres in embryos exposed to normoxia (B), and embryos exposed to 30 minutes of anoxia (F, J). Embryos exposed to 6 hours of anoxia have detectible PhosH3 in metaphase blastomeres but not prophase blastomeres (N). The letters P or M are near a representative prophase and metaphase blastomeres respectively with arrows pointed to the representative mitotic chromosomes detected by PhosH3. Scale bar equals 5 μM.

The altered chromosome localization in prophase blastomeres of embryos exposed to anoxia raises the possibility that post-translational modifications of histones control this chromosome change. Histones are subject to a variety of modifications including phosphorylation, which can be detected in *C. elegans *using indirect immunofluorescence [33]. Previously it was shown that the ability to detect phosphorylation of histone H3 at serine 10 decreased in embryos exposed to 24 hours of anoxia. However, if the embryos were allowed to recover in air for 1 hour the phosphorylated form of histone H3 was detected [7]. To determine if the dephosphorylated state of histone H3 correlates with prophase chromosome alignment to the nuclear membrane, mitotic blastomeres of embryos exposed to 30 minutes, 6, 12, 24, or 72 hours of anoxia, were fixed and stained with the DNA binding dye DAPI and the antibody that recognizes the serine 10 phosphorylation of histone H3 (PhosH3). Figure 4 shows that embryos exposed to 30 minutes of anoxia contain prophase blastomeres with phosphorylated histone H3. Phosphorylated histone H3 was decreased in prophase blastomeres of embryos exposed to 6 hours or more of anoxia yet was detectable in metaphase blastomeres (Figure 4 M–P). Similar to previously published results the phosphorylated histone H3 was reduced in metaphase and prophase blastomeres of embryos exposed to 24 hours or more hours of anoxia (data not shown) [7]. Thus, the early anoxia response of prophase chromosome alignment near the nuclear membrane does not correlate with histone H3 dephosphorylation. Rather, the histone H3 dephosphorylation is associated with prolonged anoxia exposure.

### SAN-1 is required for brief periods of anoxia

**Figure 5:**
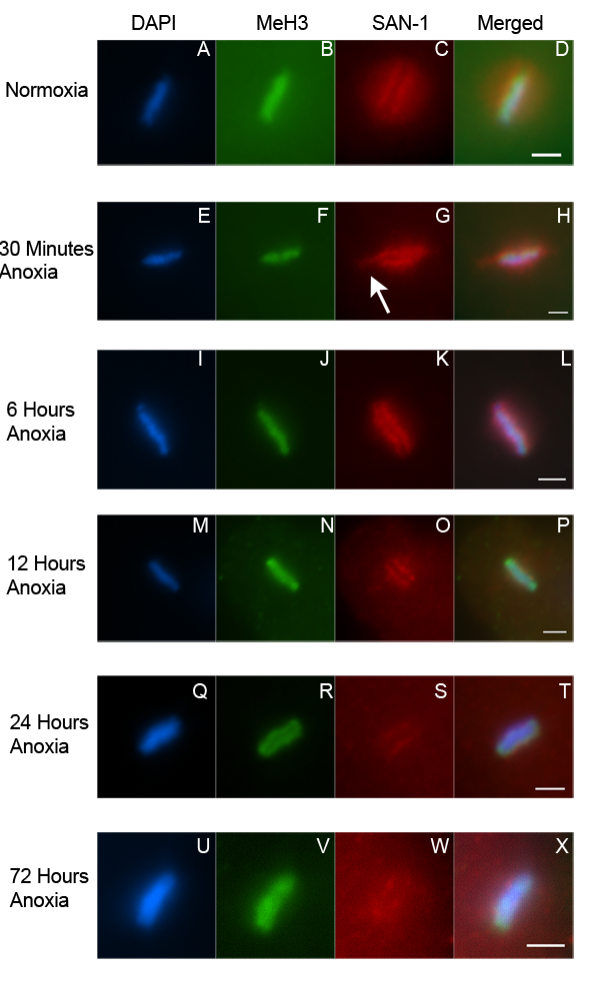
**SAN-1 localization to the kinetochore of metaphase blastomeres is reduced in embryos exposed to prolonged anoxia. **Enlarged image of metaphase blastomeres of 4–8 cell embryos exposed to either normoxia (A-D), 30 minutes (E-H), 6 hours (I-L), 12 hours (M-P), 24 hours (Q-T), or 72 hours (U-X) of anoxia, collected, and stained with DAPI (A, E, I, M, Q, U), antibody MeH3, which recognizes methylated Histone H3 (B, F, J, N, R, V), and an antibody that recognizes SAN-1 (C, G, K, O, S, W). Merged image for each set is shown (D, H, L, P, T, X). White arrow points to the lateral flare detected in metaphase blastomeres of 4-cell embryos exposed to anoxia. For all images the scale bar equals 2 μm.

An alteration in the chromosome structure of metaphase blastomeres of embryos exposed to anoxia may be due to other changes in the nucleus such as at the kinetochore or spindle. *C. elegans *chromosomes are holocentric in nature and thus contain kinetochore and microtubule attachment sites along the entire length of the chromosome [34,35]. SAN-1 is a kinetochore localized spindle checkpoint protein that is required for anoxia survival [21]. Others have shown that 4-cell embryos exposed to 24 hours of anoxia contain metaphase blastomeres with kinetochore rearrangements or "flares" that project out laterally from the metaphase plate. These lateral projections were detected whether one observed the metaphase plate from a face-on-view or the poleward facing view [28]. To gain a greater understanding of how anoxia affects SAN-1 sub-cellular localization, young embryos were exposed to anoxia for 30 minutes, 6, 12, 24 or 72 hours and stained with the DNA binding dye DAPI and an antibody to detect SAN-1 (Figure 5). In all experiments, the antibody that detects the dimethylated Lysine 4 (MeH3) was used as an antibody accessibility control, because the localization of this antigen is not altered in mitotic blastomeres of embryos exposed to anoxia. We confirm that 4-cell embryos exposed to anoxia contain detectable SAN-1 localized to the kinetochore and in lateral projections from the metaphase plate (Figure 5G). We also find that there is less detectable SAN-1 in the nucleoplasm in metaphase blastomeres of embryos exposed to 30 minutes or 6 hours of anoxia in comparison to normoxic embryos (Figure 5C, G, K). The lateral projections were not as easily detected in 8-cell embryos exposed to 6 hours or more of anoxia (Figure 5K, O, S, W) suggesting that either the kinetochore projections are only a characteristic of 4-cell embryos or the projections are more difficult to detect in embryos developed beyond the 4-cell stage. Embryos exposed to 12 hours or more of anoxia contains SAN-1 localized in punctate form in the nucleoplasm surrounding the metaphase plate (Figure 5O, S, W). Embryos exposed to at least 24 hours of anoxia display a reduction in the detection of SAN-1 at the kinetochore (Figure 5S, W). Taken together, it is concluded that SAN-1 localization changes are dependent upon anoxia exposure time.

Previously, it was shown that embryos exposed to 24 hours of anoxia contain blastomeres that arrest in interphase, prophase, metaphase and to a lesser extent telophase [7]. We have determined that embryos exposed to 30 minutes of anoxia contain blastomeres arrested at interphase and mitosis. The stages of mitosis were determined for wild-type embryos exposed to normoxic or anoxic conditions (Table 2). Results indicate that cell cycle arrest is an early response to anoxia, thus the gene products required for cell cycle arrest are likely required at initial exposure to anoxia.

**Table 2:** Mitotic stage of wild-type and *san-1(RNAi) *embryos exposed to 30 minutes of anoxia.

	Normoxia Wild-type (%, n = 213)	Normoxia *san-1(RNAi) *(%, n = 353)	Anoxia Wild-type (%, n = 256)	Anoxia *san-1(RNAi) *(%, n = 309)
Prophase	49.77	59.49	59.38	68.28
Prometaphase	10.80	5.67	4.30	0.65
Metaphase	20.19	17.28	35.94	0.97
Anaphase	9.86	9.63	0.00	0.00
Telophase	9.39	7.93	0.39	1.29
Abnormal Nuclei	0.00	0.00	0.0	26.86
Anaphase Bridges	0.00	0.00	0.0	2.59

n = total number of mitotic blastomeres assayed from at least 50 embryos of two independent experiments.

**Figure 6:**
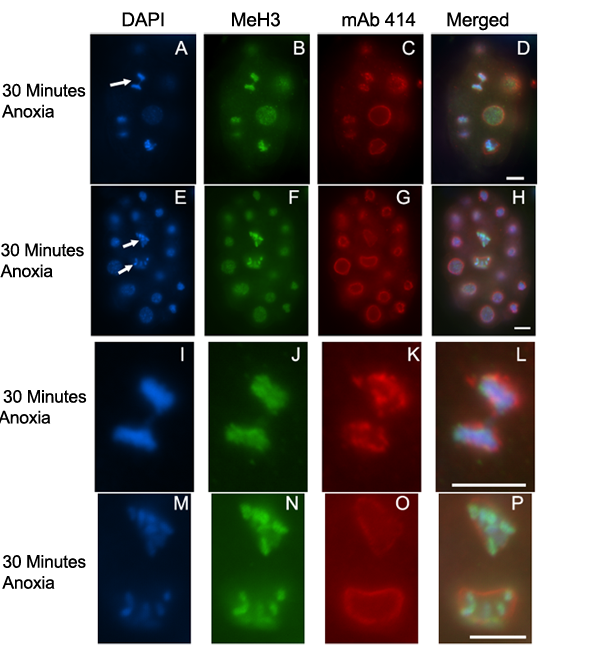
***san-1(RNAi) *embryos exposed to 30 minutes of anoxia display chromosome segregation abnormalities. **Two representative embryos that were exposed to 30 minutes of anoxia and stained with DAPI (A, E, I, M), antibody MeH3, which recognizes methylated Histone H3 (B, F, J, N), and mAb414 to detect the nuclear pore complex (C, G, K, O). Merged image for each set is shown (D, H, L, P). Arrows point to representative anaphase bridging (A) and abnormal chromatin structure (E) with the enlarged images shown in I-L and M-P, respectively. Scale bar equals 5 μm.

To determine if SAN-1 is required for brief periods of anoxia we exposed *san-1(RNAi) *embryos to 30 minutes of anoxia and determined mitotic stage and presence of anaphase bridges or abnormal nuclei (Table 2). In comparison to *san-1(RNAi) *embryos exposed to normoxia, the *san-1(RNAi) *embryos exposed to anoxia display a decrease in metaphase blastomeres and an increase in anaphase bridging and abnormal nuclei. Figure 6 shows two representative *san-1(RNAi) *embryos exposed to 30 minutes of anoxia that display abnormal nuclei and anaphase bridging. Given that this phenotype is seen in embryos exposed to just 30 minutes of anoxia indicates that SAN-1 is required for the early response to anoxia. The *san-1(RNAi) *embryos exposed to 30 minutes of anoxia contained prophase and interphase blastomeres, further supporting the idea that SAN-1 is specifically required for the arrest of metaphase blastomeres. Considering that SAN-1 is required for brief periods of anoxia and the SAN-1 localization to the kinetochore in wild-type embryos exposed to brief periods of anoxia, it is likely that the SAN-1 activity required for metaphase arrest occurs at either the kinetochore and/or at the kinetochore lateral projections. To evaluate the efficiency of RNAi technique mitotic nuclei of *san-1(RNAi) *embryos were assayed for the presence of SAN-1 localization by indirect immunofluorescence. Briefly, 82.32% (n = 141) of the mitotic blastomeres of *san-1(RNAi) *embryos were absent for SAN-1, whereas 1.54 % (n = 65) of wild-type embryos were absent for SAN-1, indicating that SAN-1 levels were reduced using the RNAi technique.

### Embryos exposed to long-term anoxia contain blastomeres with astral and spindle microtubule depolymerization

**Figure 7:**
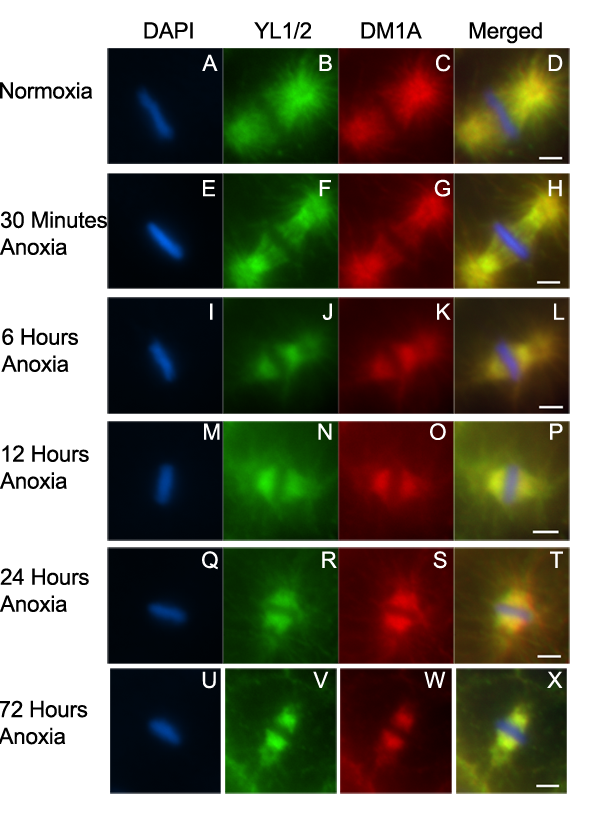
**Microtubules depolymerize in embryos exposed to anoxia. **Metaphase blastomeres were examined from embryos exposed to either normoxia (A-D), or anoxia for 30 minutes (E-H), 6 hours (I-L), 12 hours (M-P), 24 hours (Q-T) or 72 hours (U-X). Embryos were stained with DAPI (A, E, I, M, Q, U), and antibodies that recognize microtubule protein YL1/2 (B, F, J, N, R, V), and DM1A (C, G, K, O, S, W). Images were merged (D, H, L, P, T, X). Scale bar equals 2 μM.

The spindle checkpoint components monitor kinetochore-microtubule attachment and tension and act to delay the metaphase to anaphase transition until all chromatids are attached to microtubules, thus ensuring the proper segregation of chromosomes [26,27]. It is possible that a reduction in oxygen levels could affect microtubule-kinetochore attachment, thus initiating the spindle checkpoint activity of SAN-1. We used indirect immunofluorescent assays to analyze the microtubule structure of embryos exposed to various periods of anoxia. Embryos exposed to 30 minutes, 6, 12, 24, or 72 hours of anoxia, were fixed and stained with the DNA binding dye DAPI and antibodies that recognize alpha tubulin. We used two antibodies to detect microtubules in embryos; YL1/2 antibody that is known to recognize the tyrosinated form of alpha tubulin in many systems, and DM1A antibody that is known to recognize the conserved block of alpha tubulin in microtubules [36-39]. Figure 7 shows representative metaphase blastomeres of embryos exposed to anoxia. Embryos exposed to 30 minutes of anoxia contain detectable spindle microtubules, however in comparison to wild-type embryos the density of the spindle and astral microtubules appears reduced suggesting that microtubule depolymerization occurs in embryos exposed to brief periods of anoxia (Figure 7A–H). As embryos were exposed to increased anoxia exposure times the degree of astral and spindle microtubule depolymerization increased and the depolymerization of astral microtubules appeared more extensive in comparison to the spindle depolymerization. The embryos that were exposed to 72 hours of anoxia exhibited the highest degree of microtubule depolymerization (Figure 7U–X). Embryos that were exposed to 30 minutes of anoxia and then exposed to air, for as little as six minutes, before fixation did not display the reduced spindle microtubule density, indicating that microtubule polymerization could quickly recover after re-exposure to air (data not shown). In general the localization of microtubules detected with either YL1/2 or DM1A overlapped, suggesting that tyrosination of alpha tubulin is not altered in anoxia embryos.

To determine if the spindle microtubule depolymerization affected the size of the spindle structure, we calculated the average perimeter surrounding the spindle structure, in a single plane of focus, in metaphase blastomeres of embryos exposed to either normoxia or anoxia. We found that embryos exposed to 6 hours or more of anoxia had a significantly reduced spindle structure perimeter in comparison to that in normoxic embryos and the perimeter of the spindle microtubules decreased as anoxia exposure time increased (Table 1). The spindle microtubules located near the outer edge of the metaphase plate appeared less abundant in comparison to other regions of the metaphase plate. The viability of embryos exposed to 3 days of anoxia is over 90%, thus the microtubule depolymerization does not significantly reduce the survival rate.

## Discussion

Given that *C. elegans *is a soil nematode, it is subjected to bouts of oxygen deprivation in its natural environment and thus has likely adapted survival mechanisms. The *C. elegans *embryo responds to anoxia by entering into a state of suspended animation in which developmental and cell cycle progression arrests until oxygen is reintroduced into the environment. Embryos exposed to either brief periods (30 minutes) or longer periods (24 hours) of anoxia contain blastomeres that arrest at interphase, prophase, and metaphase, indicating that cell cycle arrest is an immediate and maintained response to anoxia. To gain a greater understanding of arrested blastomeres we took a cell biological approach and analyzed the sub-nuclear structures.

### Prophase blastomeres of embryos exposed to anoxia

The majority of mitotic checkpoint research focuses on the spindle checkpoint, however there is some evidence that a prophase checkpoint exists. For example, in human cells the regulation of entry into metaphase involves CHFR [40]. Theoretically, in the *C. elegans *embryo exposed to anoxia a prophase checkpoint could be activated to halt progression from prophase to metaphase. The mechanism of arresting blastomeres in prophase is not yet understood however we hypothesize that the mechanism would become activated immediately after anoxia exposure.

Prophase blastomeres of embryos exposed to anoxia contain chromosomes aligned near the nuclear membrane regardless of anoxia exposure time. Others have demonstrated that *Drosophila *embryos exposed to 24 hours of anoxia also have prophase chromosomes within the vicinity of the nuclear membrane indicating that prophase chromosome alignment with the nuclear membrane is a conserved response to oxygen deprivation [10]. However, the functional role of this response to anoxia is not known. It has been proposed that the attachment of chromatin to nuclear substructures, such as the nucleolus, nuclear periphery proteins, or the nuclear matrix may restrict chromatin movement [41]. It is thought that chromatin mobility can be regulated by the density of attachment sites [42] and that genomic regions associated with the nuclear periphery display significantly more constrained movements than other, more nucleoplasmic loci [43]. Thus, it is a possibility that one or more of these nuclear substructures is involved with the signal of the prophase chromosomes to translocate to the nuclear membrane region. However, further studies are required to understand the mechanism and functional role of prophase chromosome association near the nuclear membrane in response to anoxia.

Embryos exposed to brief periods of anoxia (30 minutes) contain prophase blastomeres with detectible phosphorylated histone H3 and embryos exposed to longer periods of anoxia (6 or more hours) do not contain prophase blastomeres with detectible phosphorylated histone H3. This suggests that dephosphorylation of histone H3 in prophase blastomeres is not associated with the initiation of anoxia-induced suspended animation. Furthermore, the observation that prophase chromosome association near the nuclear membrane precedes the detectible dephosphorylation of histone H3 indicates that chromosome alignment near the nuclear membrane occurs regardless of histone H3 phosphorylation.

### Metaphase blastomeres of embryos exposed to anoxia

Embryos exposed to 6 hours of anoxia contain prophase blastomeres with dephosphorylated histone H3 and metaphase blastomeres with phosphorylated histone H3. There are several possible explanations as to why this heterogeneity is observed. First, perhaps the phosphorylated form of histone H3 along the chromosomes is gradually decreasing and is not functioning as an all-on or all-off phenomenon. Second, the detection of phosphorylated histone H3 may be a factor of the concentration of phosphorylated histone H3 per unit space. That is, metaphase chromosomes may be more condensed and/or in very close proximity to one another, at the equatorial plate, in comparison to prophase chromosomes. Thus, in comparison to prophase blastomeres, the metaphase blastomeres may contain more phosphorylated histone H3 per unit space, resulting in a better ability to detect the signal. Third, the mechanisms involved with dephosphorylation of Histone H3 are more active or have better access to the histone H3 in prophase blastomeres. Regardless, we show that the detection of histone H3 phosophorylated state is reduced as embryos are exposed to longer periods of anoxia.

Embryos exposed to brief periods of anoxia (30 minutes) contain metaphase blastomeres in which SAN-1 is associated with the kinetochore or lateral projections and there is a reduction in astral and spindle microtubule structure. Increased anoxia exposure times result in a further reduction of spindle microtubule structure, reduction in SAN-1 localized to the kinetochore, reduction in the metaphase plate length and increase in the metaphase plate width. The relationships between these characteristics are not understood, but data indicates that SAN-1 localization changes and microtubule depolymerization precede the metaphase plate size changes.

Embryos exposed to brief periods of anoxia (30 minutes) require the spindle checkpoint protein SAN-1. The mechanism by which a decrease in oxygen signals the activity of SAN-1 is not completely understood. One possibility for spindle checkpoint protein activation is through a reduction in the kinetochore/microtubule association and/or microtubule tension. The activation of spindle checkpoint proteins such as SAN-1 could then inhibit the anaphase-promoting complex, thus arresting the metaphase blastomeres in metaphase. Further genetic and cellular analyses are required to test this model and to determine how a reduction in oxygen signals the depolymerization of microtubules.

### Interphase blastomeres of embryos exposed to anoxia

Embryos exposed to brief periods of anoxia (30 minutes) contain interphase blastomeres with condensed chromatin. Embryos exposed to longer periods of anoxia (24 hours) contain interphase blastomeres with increased chromatin condensation and chromatin associated near the nuclear membrane. Thus, one model is that a reduction in oxygen induces a reorganization and condensation of chromatin in the nucleus, which could in turn reduce and control energy requiring processes such as gene expression. The signal between anoxia and the reorganization of chromatin is not yet understood, but it would be of interest to determine if the reorganization of chromatin is involved with the arrest of blastomeres in interphase.

Humans are less capable of surviving oxygen deprivation in comparison to other organisms. Thus, understanding the cellular responses to oxygen deprivation in oxygen deprivation tolerant organisms such as *C. elegans *is of value. Some of the responses to oxygen deprivation are conserved between *C. elegans *and humans. For example, the hypoxia inducing factor (HIF-1) is important for sensing and surviving low oxygen (hypoxia) in humans and various model systems including *C. elegans *[44,45]. However, in *C. elegans*, HIF-1 is not required for anoxia survival suggesting that alternate oxygen sensing pathways are present [7]. The characterization of the cellular response to anoxia will lead to identification of additional oxygen sensing pathways as well as deciphering additional mechanisms required for oxygen deprivation survival.

## Conclusion

In this report we describe the sub-nuclear characteristics of prophase, metaphase and interphase blastomeres of embryos exposed to anoxia for various time periods. A summary of the sub-nuclear changes associated with anoxia is presented in Table 3. The data indicates that sub-cellular changes that occur in embryos exposed to anoxia are dependent upon anoxia exposure time and cell cycle stage. Since cell cycle arrest is an event that occurs early upon anoxia exposure, we hypothesize that anoxia-induced cell cycle arrest is controlled by changes that occur during the initial exposures to anoxia. Our data shows that the initial events that occur in arrested metaphase blastomeres include spindle microtubule changes and SAN-1 localization changes. Additionally, SAN-1 is required for proper arrest of metaphase blastomeres of embryos exposed to brief periods of anoxia. Thus, we hypothesize that either spindle microtubule depolymerization and/or SAN-1 localization changes control anoxia-induced metaphase arrest. The mechanisms that control prophase and interphase blastomere arrest are not understood, but we suggest that the mechanism must be activated early upon anoxia exposure. So, in regards to prophase blastomeres, we hypothesize that an initial event such as prophase chromosome alignment to the nuclear membrane could be involved with the proper arrest of prophase blastomeres. In regards to interphase blastomeres, we hypothesize that an initial event such as chromatin condensation may be important for arrest of interphase blastomeres. Further genetic analysis is required to test these hypotheses. The work presented here builds a foundation for understanding how oxygen deprivation controls cell cycle arrest and progression.

**Table 3:** Summary of the Sub-cellular Characteristics of Embryos Exposed to Anoxia.

Normoxia	30 Minutes Anoxia (Brief)	6–12 Hours Anoxia (Intermediate)	24–72 Hours Anoxia (Long-term)
**Prophase Blastomeres:**			
Chromosomes Throughout Nucleoplasm	Chromosomes Aligned Near Nuclear Membrane	Chromosomes Aligned Near Nuclear Membrane	Chromosomes Aligned Near Nuclear Membrane
Histone H3 Phosphorylation	Histone H3 Phosphorylation	Histone H3 Dephosphorylation	Histone H3 Dephosphorylation
**Metaphase Blastomeres:**			
Metaphase Plate Normal	Metaphase Plate Normal	Metaphase Plate LengthReduced	Metaphase Plate LengthReduced
Histone H3 Phosphorylation	Histone H3 Phosphorylation	Histone H3 Phosphorylation	Histone H3 Dephosphorylation
Microtubules Polymerization	Detectable Microtubule Depolymerization	Microtubule Spindle Perimeter Reduced	Microtubule Spindle Perimeter Reduced
SAN-1 Localizationat Kinetochore and Nucleoplasm	SAN-1 Localization at Kinetochore or Lateral Extensions	SAN-1 Localization at Kinetochore or Lateral Extensions	SAN-1 Reduced Localization at Kinetochore
SAN-1 not essential	SAN-1 essential	SAN-1 essential	SAN-1 essential
**Interphase Blastomeres:**			
Chromosomes Throughout Nucleoplasm	Chromosome Condensation	Chromosome Condensation and Alignment Near Nuclear Membrane	Chromosome Condensation and Alignment Near Nuclear Membrane

## Methods

### Strains and growth conditions

Bristol strain N2 was cultured on NGM as described [46]. AZ212 strain (*unc-119(ed3) ruIs32 [unc-119(+) pie-1*::GFP::H2B] III) was obtained from the *Caenorhabditis elegans *Genetics Center [29].

### Oxygen deprivation experiments

Synchronized populations of nematodes were obtained as embryos from hypochlorite treated adults and were allowed to develop to young hermaphrodites with eggs in them. The gravid adults were exposed to an anoxic environment at 20°C for 30 minutes, 6, 12, 24, or 72 hours using the BioBag Type A anoxia chamber (Becton and Dickinson, Cockeysville, MD)[11]. The adults were then removed from the BioBag Type A chamber and minced within an anoxia glove box workstation (Invivo_2 _200 anoxia/hypoxia workstation, Biotrace International, Cincinnati, OH) and eggs were collected onto a slide and placed on dry ice. For all experiments embryos exposed to anoxia were prepared for fixation in the anoxia glove box chamber to ensure the analysis of anoxic responses and not the analysis of post-anoxic recovery due to brief exposures to normoxia during embryo preparation. For post-anoxic experiments, embryos were allowed to recover in air, for the time indicated, prior to analysis. Embryos from normoxic control adults were collected when the environment, for the experimental embryos, transitioned from normoxia to anoxia, which was approximately one hour after placement into the BioBag type A anoxia chamber. For all studies the anaerobic BioBag type A environmental chamber and the Invivo_2 _200 anoxia/hypoxia glove box chamber were used according to manufacturers instructions. Briefly, the anaerobic BioBag type A environmental chamber uses potassium borohydride, sodium bicarbonate, hydrochloric acid, and a palladium catalyst cup to generate an anaerobic environment in which resazurin is used as an oxygen indicator. The Invivo_2 _200 anoxia/hypoxia glove box chamber has a gas mixing system that produced an anaerobic environment (5% Carbon Dioxide, 0% Oxygen, 10% Hydrogen, balanced with Nitrogen).

### Indirect immunofluorescence and image analysis

Embryos were prepared as described by [7,35,37]. Briefly, embryos were immediately placed on slides and onto dry ice following anoxic treatment. Embryos were freeze cracked and fixed in either N,N- dimethylformamide or methanol for 5 minutes at -20°C, followed by a 5 minute incubation in PBS. Embryos were incubated for 30 minutes in PBS Block (3% BSA, .1% Tween 20 and 2 mM MgCl_2_) followed by a 1-hour primary antibody incubation, two washes with PBS Block, a 30 minute incubation with the appropriate fluorescently labelled (FITC or TRITC) secondary antibody incubation (Jackson ImmunoResearch Laboratories) and two washes with PBS block, a 5 minute incubation with .05 mg/ml DAPI (4',6-diamidino-3-phenylindolle dihydrochloride) followed by use of prolong anti-fading reagent to reduce bleaching (Molecular Probes, Eugene, OR). For microtubule detection, embryos were fixed in 100% Methanol at -20°C for 5 minutes, followed by the staining procedure described above.

The following primary antibodies were used for these studies: rabbit anti-Phos H3 to detect the phosphorylated (Ser10) form of histone H3 (Update Biotechnology, Lake Placid, NY); mAb414 to detect nuclear envelope pore complexes (BabCo, Richmond, CA), mouse anti-SAN-1 to detect SAN-1 [21]; mAb DM1A (Sigma-Aldrich, St. Louis, MO) YL1/2 (AbCam, Cambridge MA) to detect alpha tubulin, MetH3 to detect the methylated form of Histone H3 (BabCo, Richmond, CA), anti-gamma tubulin to detect the centrosome (Sigma-Aldrich, St. Louis, MO). The antibodies were used in a working dilution of 1:500 or 1:200 in the case of anti-gamma tubulin.

Embryos evaluated were primarily early in development (2-cell to 50-cell stage), thus allowing better resolution of cellular structures. Embryos were visualized using Zeiss Axioscope mot plus 2, with a 100X/1.3 oil plan-neofluar objective lens. Images were captured and processed using the camera Zeiss Axiocam camera and Openlab software 3.1.7 (Improvision Inc, Lexington, MA). Exposure time was similar for experimental and control embryos. For all indirect immunofluorescent protein localization studies at least three independent experiments were conducted. Prophase blastomeres of embryos were evaluated using a Perkin Elmer Ultraview ERS spinning disk confocal microscope. Image acquisition and visualization was carried out using software provided with Ultraview ERS developed by PerkinElmer. Image processing and analysis was conducted using the software package Image J from NIH. For image demonstration of the cross sections images were imported into QuickTime 7 Pro (Apple Computer, Inc.).

### Analysis of metaphase plate and spindle structure

Embryos, at similar developmental stages (< 20 blastomeres) were exposed to either normoxic or anoxic conditions and prepared for indirect immunofluorescence as described above. Embryos were fixed and stained with DAPI. The metaphase plate is "disc shaped" thus, to determine the length of the metaphase plate, which is the diameter, the focal plane in which the diameter was largest was used for calculations. The width of the metaphase plate was determined at the same focal plane that the diameter was determined. The metaphase plate length and width was calculated using the imaging software program Openlab 3.1.7. This software was also used to draw around the spindle microtubule structure to calculate the perimeter of the spindle structure, associated with one set of sister chromatids, in metaphase blastomeres. The software allows one to calculate the number of micrometers a specific line drawing is.

### Live imaging

Synchronized gravid AZ212 (*pie-1*::GFP::H2B) adults were placed in either a normoxic or anoxic environment (6 hours or 12 hours). The AZ212 strain contains the GFP::H2B fusion protein, regulated by the *pie-1 *promoter, thus allowing chromosomes to be analyzed in live embryos. Adults were removed, chopped and embryos were collected and placed onto a 2.5 % agar pad for microscopy imaging [46]. For all experiments, time from removal from anoxia to time of imaging was 15 minutes. Embryos were visualized using a motorized Zeiss Axioscope mot plus 2 microscope and the programmable automation Openlab software 3.1.7. Differential interference contrast (DIC) and GFP images were acquired every 30 seconds for 20 minutes. These time-lapsed images were used to quantify the mitotic progression time of blastomeres in embryos exposed to anoxia. The stages of mitosis were characterized as follows: prophase: the chromosomes began to condense; prometaphase: the condensed chromosomes began to move to the equatorial plate of the blastomere; metaphase: the chromosomes were aligned at the equatorial plate; anaphase: the chromosomes began to segregate until the segregating chromosomes no longer moved and took on a round shape.

### RNAi experiments

The method used for *san-1(RNAi) *is previously described [21]. Briefly, synchronous population of L1 worms were grown to adulthood on NGM plates supplemented with 200 μg/ml Ampicillin, 12.5 μg/ml tetracycline and 2 mM IPTG, seeded with bacterial strain expressing dsRNA specific for *san-1 *[47,48]. Adults were placed into an anoxic environment and embryos were removed from the adult and prepared for indirect immunofluorescence analysis as described above. To evaluate the efficiency of RNAi technique for depleting SAN-1, indirect immunofluorescent assays of *san-1(RNAi) *embryos were performed. Control embryos and *san-1(RNAi) *embryos were stained with anti-SAN-1 and Met H3, to assay for the presence or absence of SAN-1 in mitotic blastomeres and as an accessibility control, respectively.

## Authors' contributions

VAH participated in the design of the study, conducted the indirect immunofluorescent assays and assisted in the drafting of the manuscript.

JT conducted the analysis of embryos recovering from anoxia and assisted in the manuscript preparation.

PAP conceived the study, participated in the design and coordination of the study, assisted in the immunofluorescent assays and drafted the manuscript.

All authors read and approved the final manuscript.

## Supplementary Material

Additional File 1 Chromosomes of prophase nucleus of an embryo exposed to normoxia. Embryos were collected, and stained with DAPI and the mAb414 to recognize nuclear pore complex. Spinning disk confocal microscopy was used to obtain Z-stack images of the prophase nucleus. The movie (QuickTime Player 7.0.3) depicts a 9.2 μm region that includes the nucleus cross-sectioned every 0.2 μm for a total of 46 cross sections. DAPI and mAb414 images were merged to evaluate prophase chromosome location relative to the nuclear membrane. Click here for file

Additional File 2 Chromosomes of prophase nucleus of an embryo exposed 30 minutes of anoxia. Embryos were collected, and stained with DAPI and the mAb414 to recognize nuclear pore complex. Spinning disk confocal microscopy was used to obtain Z-stack images of the prophase nucleus. The movie (QuickTime Player 7.0.3) depicts a 9.2 μm region that includes the nucleus cross-sectioned every 0.1 μm for a total of 92 cross sections. DAPI and mAb414 images were merged to evaluate prophase chromosome location relative to the nuclear membrane. Click here for file

Additional File 3 Chromosomes of prophase nucleus of an embryo exposed 24 hours of anoxia. Embryos were collected, and stained with DAPI and the mAb414 to recognize nuclear pore complex. Spinning disk confocal microscopy was used to obtain Z-stack images of the prophase nucleus. The movie (QuickTime Player 7.0.3) depicts an 8.4 μm region that includes the nucleus cross-sectioned every 0.2 μm for a total of 42 cross sections. DAPI and mAb414 images were merged to evaluate prophase chromosome location relative to the nuclear membrane. Click here for file

Additional File 4 Chromosomes of prophase nucleus of an embryo exposed to normoxia. Embryos were collected, and stained with DAPI, mAb414 to recognize nuclear pore complex and anti-gamma tubulin to recognize the centrosome. Spinning disk confocal microscopy was used to obtain Z-stack images of the prophase nucleus. The movie (QuickTime Player 7.0.3) depicts a 10.0 μm region that includes the nucleus cross-sectioned every 0.2 μm for a total of 50 cross sections. DAPI, mAb414 and anti-gamma tubulin images were merged to evaluate prophase chromosome location relative to the nuclear membrane. Click here for file

Additional File 5 Chromosomes of prophase nucleus of an embryo exposed 30 minutes of anoxia. Embryos were collected, and stained with DAPI, mAb414 to recognize nuclear pore complex and anti-gamma tubulin to recognize the centrosome. Spinning disk confocal microscopy was used to obtain Z-stack images of the prophase nucleus. The movie (QuickTime Player 7.0.3) depicts a 7.0 μm region that includes the nucleus cross-sectioned every 0.2 μm for a total of 35 cross sections. DAPI, mAb414 and anti-gamma tubulin images were merged to evaluate prophase chromosome location relative to the nuclear membrane. Click here for file
